# Etiological analysis of infection after CRS + HIPEC in patients with PMP

**DOI:** 10.1186/s12885-023-11404-1

**Published:** 2023-09-26

**Authors:** Rui Yang, Xin Zhao, Yu-Bin Fu, Yu-Lin Lin, Ru Ma, Yan-Dong Su, He-Liang Wu, Xin-Li Liang, Yan Li

**Affiliations:** 1 Department of Peritoneal Cancer Surgery, Beijing Shijitan Hospital, Capital Medical University, Haidian District, No. 10 Tieyi Road, Yangfangdian Street, Beijing, 100038 China; 2 Department of Peritoneal Cancer Surgery, Beijing Shijitan Hospital, Peking University Ninth School of Clinical Medicine, Beijing, 100038 China; 3 Department of Surgical Oncology, Beijing Tsinghua Changgung Hospital, Tsinghua University, Beijing, 102218 China

**Keywords:** PMP, CRS + HIPEC, postoperative infection, Bacteria, Antibiotic

## Abstract

**Background:**

Cytoreductive surgery (CRS) plus hyperthermic intraperitoneal chemotherapy (HIPEC) is the standard treatment for pseudomyxoma peritonei (PMP). It can significantly prolong the survival of patients, but at the same time may increase the risk of postoperative infection.

**Method:**

Patients with PMP who underwent CRS + HIPEC at our center were retrospectively analyzed. According to PMP patients**,** basic clinical data and relevant information of postoperative infection, we analyzed the common sites of postoperative infection, results of microbial culture and the antibiotics sensitivity. Univariate and multivariate analysis were performed to explore infection-related risk factors.

**Result:**

Among the 482 patients with PMP, 82 (17.0%) patients were infected after CRS + HIPEC. The most common postoperative infection was central venous catheter (CVC) infection (8.1%), followed by abdominal-pelvic infection (5.2%). There were 29 kinds of microbes isolated from the culture (the most common was *Staphylococcus epidermidis*), including 13 kinds of Gram-positive bacteria, 12 kinds of Gram-negative bacteria, and 4 kinds of funguses. All the antibiotics sensitivity results showed that the most sensitive antibiotics were vancomycin to Gram-positive bacteria (98.4%), levofloxacin to Gram-negative bacteria (68.5%), and fluconazole to fungus (83.3%). Univariate and multivariate analysis revealed the infection independent risk factors as follow: intraoperative blood loss ≥ 350 mL (*P* = 0.019), ascites volume ≥ 300 mL (*P* = 0.008).

**Conclusion:**

PMP patients may have increased infection risk after CRS + HIPEC, especially CVC, abdominal-pelvic and pulmonary infections. The microbial spectrum and antibiotics sensitivity results could help clinicians to take prompt prophylactic and therapeutic approaches against postoperative infection for PMP patients.

## Introduction

Pseudomyxoma peritonei (PMP) is a malignant clinical syndrome characterized by the accumulation and redistribution of mucus produced by mucinous tumor cells in the peritoneal cavity [1]. Most of PMP originate from mucinous tumors of the appendix, and a few originate from primary mucinous tumors of ovaries, colons and other organs [2]. The incidence of PMP is approximately 2–4 cases in 1 million per year [3–5], and the prevalence is approximately 25.1 cases in 1 million; the male/female ratio is 1: (1.2 to 3.4) [3, 4, 6], the median age of onset was 62–63 years [7–10].

Nowadays, the integrated treatment which focuses on CRS + HIPEC is the main strategy for the treatment of PMP [11, 12]. For the selected PMP patients, standardized CRS + HIPEC can significantly improve their overall survival up to 103.4–196 months, and the 5-year and 10-year survival rates can reach 92.1% and 80.8% [13].

CRS is a complex surgical procedure, lasts a long time, and generally has a wide range of excision, which may have a great impact on the patients. The drugs of HIPEC may expose patients to the risk of immunosuppression, and splenectomy for some patients due to local invasion will also increase the risk of immunosuppression [14]. Thus, PMP patients are potentially at high risk for postoperative infection. According to literature reports, the incidence of postoperative infection adverse events after CRS + HIPEC is about 21.0% ~ 43.0% [15–19].

This study aims to analyze the common infection sites, microbes and corresponding antibiotics sensitivity result of PMP patients after CRS + HIPEC. So as to provide reference for the treatment of patients with such postoperative infections.

## Patients and methods

### Clinical data

This study was approved by the institutional review board of Beijing Shijitan Hospital, Capital Medical University (2015-[20]). All patients signed an informed consent to receive CRS + HIPEC and for the use of their clinicopathological data for further research and academic publications.

This retrospective study included 482 patients with PMP treated with CRS + HIPEC at Beijing Shijitan Hospital from May 2015 to April 2022. Data regarding the basic clinicopathological characteristics, CRS + HIPEC related information, and postoperative infection related information (results of microbial culture and antibiotic sensitivity test, etc.) were collected.

### Patient selection

All patients met the criteria for CRS + HIPEC surgery [21], and the inclusion criteria were as follows: (1) Karnofsky performance status score > 60; (2) normal peripheral blood white blood cell count ≥ 3,500/mm^3^ and platelet count ≥ 80,000/mm^3^; (3) acceptable liver function, with total bilirubin ≤ 2 × the upper limit of normal (ULN) and aspartic aminotransferase and alanine aminotransferase ≤ 2 × ULN; (4) acceptable renal function, with serum creatinine ≤ 1.5 mg/dL; and (5) other major organ functions can tolerate a major operation. Major exclusion criteria include: (1) preoperative examination revealing distant metastases; (2) imaging examination indicating mesenteric contracture; and (3) the performance status and function of vital organs that cannot tolerate major surgery.

### CRS + HIPEC

All CRS + HIPEC procedures were performed by the peritoneal metastasis specialist team of our center. After successful general anesthesia, a midline incision was made in the upper abdomen from the xiphoid process to the pubic symphysis to expose the abdominal cavity fully. And then, peritoneal cancer index (PCI) score was comprehensively evaluated. After CRS, the completeness of cytoreduction (CC) score was evaluated based on the residual tumor size. Open HIPEC was administered after completion of CRS, with 120 mg cisplatin + docetaxel 120 mg, or 120 mg cisplatin + mitomycin 30 mg at 43℃ for 60 min. Subsequently, functional reconstruction of digestive tract and abdominal closure were performed.

### Postoperative infection

Clinical infection should be suspected in patients with postoperative symptoms such as dyspnea, painful urination, suppurative discharge in the wound or drainage tube, or fever > 38℃. Persistent increases in neutrophil counts, procalcitonin and or C-reactive protein levels 48 h after CRS + HIPEC were also considered suspected factors for infection. For patients suspected of infection, microbial culture and antibiotics sensitivity test should be carried out on any samples obtained clinically, in order to detect infection as early as possible and take targeted treatment.

#### CVC infection

CVC infection was defined as positive CVC tip microbial culture accompanied by chills and fever (> 38℃) or positive venous blood microbial culture (consistent with the results of CVC tip microbial culture) within 30 days after CRS + HIPEC.

#### Abdominal-pelvic infection

Postoperative abdominal-pelvic infection was defined as positive microbial culture of the patient's abdominal-pelvic drainage, accompanied by signs of peritonitis, fever or other infection-related symptoms within 30 days after CRS + HIPEC.

#### Pulmonary infection

Postoperative pulmonary infection was defined as positive microbial culture of the patient's sputum within 30 days after CRS + HIPEC, accompanied by imaging signs of infection or symptoms such as fever, cough and sputum.

#### Other types of infection

Postoperative surgical wound infection was defined as positive microbial culture of the patient's incision exudate within 30 days after CRS + HIPEC, accompanied by infection symptoms such as skin swelling, heat and pain around the incision. Postoperative urinary system infection was defined as positive microbial culture of midstream urine accompanied by urinary tract irritation or systemic infection symptoms such as fever and chills within 30 days after CRS + HIPEC. Postoperative positive blood culture of bacteria/fungi (infection site unknown) was defined as that the patients showed systemic infection symptoms such as fever and chills within 30 days after CRS + HIPEC with the blood culture of microbe was positive, but the infection site was still unclear after various examinations and physical examination.

### Statistical analysis

Microsoft Excel 2016 and IBM SPSS Statistics for Windows, version 26.0 were used for data analysis. Measurement data were presented as median (range) or mean ± SD and analyzed by t-test or rank-sum test. Enumeration data were presented as frequencies and analyzed using the χ2 and Fisher’s exact tests. Univariate and logistic regression analysis were used to analyze the independent factors influencing postoperative infection. The Kaplan–Meier method and log-rank test were used for survival analysis. Statistical significance was set at *P* < 0.05.

### Overall survival

Overall survival (OS) was defined as the time interval from the date of clinical diagnosis to the date of death or last follow-up.

## Result

### Major clinicopathologic characteristics

A total of 482 patients with PMP were included in this study, including 211 males (43.8%) and 271 females (56.2%). The median age was 55 (25–75) years. Pathological diagnosis showed that there were 15 (3.1%) cases of acellular mucus, 268 (55.6%) cases of low grade, 153 (31.7%) cases of high grade, and 46 (9.5%) cases of high grade with signet-ring cells. There were 31 (6.4%) patients with vascular tumor thrombus and 32 (6.6%) patients with lymphatic invasion. The median duration of CRS + HIPEC operation was 640 (95–1,082) minutes. The median number of organs resected and stripped peritoneum area were 3 (0–10) and 5 (0–9), respectively. The median PCI score was 29 (1–39), and 258 (53.5%) cases achieved CC0-1. Anastomosis was found in 350 patients (72.6%). The main clinicopathological characteristics and surgical parameters are shown in Table 1.

**Table 1:** Clinicopathological characteristics and CRS + HIPEC parameters of patients with PMP

Variables	Value
Gender, n (%)	
Male	211(43.8)
Female	271(56.2)
Age (years), median (range)	55(25–75)
Intravenous chemotherapy, n (%)	
No	298(61.8)
Yes	184(38.2)
KPS, median (range)	90(60–100)
BMI (kg/m2), median (range)	23.0(15.2–40.0)
Pathological diagnosis, n (%)	
Acellular mucus	15(3.1)
Low grade	268(55.6)
High grade	153(31.7)
High grade with signet ring cells	46(9.5)
Vascular invasion, n (%)	
No	451(93.6)
Yes	31(6.4)
Lymphatic metastasis, n (%)	
No	450(93.4)
Yes	32(6.6)
Operative duration (min), median (range)	640(95–1,080)
hospital length of stay (d), median (range)	24(11–97)
Resected organs, median (range)	3(0–10)
Stripped peritoneum area, median (range)	5(0–9)
Anastomosis, n (%)	
No	132(27.4)
Yes	350(72.6)
PCI, median (range)	29(1–39)
CC, n (%)	258(53.5)
0–1	224(46.5)
2–3	600(20–5,000)
Intraoperative blood loss (mL), median (range)	3.5(0–20)
RBC transfusion volume (U), median (range)	800(0–2,000)
Plasma transfusion volume (mL), median (range)	500(0–20,000)
Ascites volume (mL), median (range)	258(53.5)

*BMI* Body mass index, *KPS* Karnofsky performance status, *PCI* Peritoneal cancer index, *CC* Completeness of cytoreduction, *RBC* Red blood cells

### Thesites of postoperative infection

Among the 482 patients with PMP, 82 (17.0%) cases had postoperative infection with no patients died due to the infection, including 39 (8.1%) cases of CVC infection, 25 (5.2%) cases of abdominal-pelvic infection, 23 (4.8%) cases of pulmonary infection, 10 (2.1%) cases of surgical wound infection, 5 (1.0%) cases of urinary system infection, and 5 (1.0%) cases of blood culture microbe positive (unknown infection site) (Table 2).

**Table 2:** Sites of postoperative infection in patients with PMP

Infection sites	Patients(n)	Accounted for the proportion of postoperative infection patients (%)	Accounts for the proportion of all patients (%)
CVC infection	39	47.6	8.1
Abdominal and pelvic infection	25	30.5	5.2
Pulmonary infection	23	28.1	4.8
Surgical wound infection	10	12.2	2.1
Urinary system infection	5	6.1	1.0
Blood culture bacteria/fungi positive (unknown infection site)	5	6.1	1.0
≥ 2 Infection sites	21	25.6	4.4

*CVC* Central venous catheter

### Types of infected microbe and drug susceptibility

#### Types of infected microbe

The 82 patients who had postoperative infection were infected with a total of 29 types of bacteria and fungi, including 13 types of Gram-positive bacteria (*Staphylococcus epidermidis*, *Staphylococcus hominis subsp. hominis*, *Staphylococcus aureus*, *Enterococcus faecalis*, *Staphylococcus capitis*, *Enterococcus faecium*, *Gemella haemolysans*, *Staphylococcus caprae*, *Streptococcus mitis*, *Streptococcus oralis*, *Streptococcus intermedius*, *Streptococcus anginosus*, and *Enterococcus casseliflavus*), 12 types of Gram-negative bacteria (*Acinetobacter baumannii*, *Klebsiella pneumoniae*, *Escherichia coli*, *Enterobacter cloacae subsp. Cloacae*, *Pseudomonas aeruginosa*, *Enterobacter aerogenes*, *Acinetobacter nosocomialis*, *Enterobacter kobei*, *Stenotrophomonas maltophilia*, *Enterobacter asburiae*, *Klebsiella aerogenes*, *Klebsiella oxytoca*), 4 types of fungi (*Candidia albicans*, *Candida parapsilosis*, *Candida famata*, *Candida tropicalis*). 50 (61.0%) patients were infected with Gram-positive bacteria, among which the most common was *Staphylococcus epidermidis* (25.6%), followed by *Staphylococcus hominis subsp. hominis* (12.2%), *Staphylococcus aureus* (11.0%). 41 patients (50.0%) were infected with Gram-negative bacteria, of which the most common was *Acinetobacter baumannii*, followed by *Klebsiella pneumoniae* (9.8%) and *Escherichia coli* (9.8%) (Table 3).

**Table 3:** The types and proportion of the microbes isolated from PMP patients with postoperative infection

Variable	Infected patients (n)	Proportion (%)
Gram-positive bacteria	50	61.0
*Staphylococcus epidermidis*	21	25.6
*Staphylococcus hominis subsp. hominis*	10	12.2
*Staphylococcus aureus*	9	11.0
*Enterococcus faecalis*	8	9.8
*Staphylococcus capitis*	2	2.4
*Enterococcus faecium*	2	2.4
*Gemella haemolysans*	1	1.2
*Staphylococcus caprae*	1	1.2
*Streptococcus mitis*	1	1.2
*Streptococcus oralis*	1	1.2
*Streptococcus intermedius*	1	1.2
*Streptococcus anginosus*	1	1.2
*Enterococcus casseliflavus*	1	1.2
Gram-negative bacteria	41	50.0
*Acinetobacter baumannii*	13	15.9
*Klebsiella pneumoniae*	8	9.8
*Escherichia coli*	8	9.8
*Enterobacter cloacae subsp. Cloacae*	7	8.5
*Pseudomonas aeruginosa*	6	7.3
*Enterobacter aerogenes*	2	2.4
*Acinetobacter nosocomialis*	1	1.2
*Enterobacter kobei*	2	2.4
*Stenotrophomonas maltophilia*	1	1.2
*Enterobacter asburiae*	1	1.2
*Klebsiella aerogenes*	1	1.2
*Klebsiella oxytoca*	1	1.2
Fungus		
*Candida albicans*	3	3.7
*Candida parapsilosis*	1	1.2
*Candida famata*	1	1.2
*Candida tropicalis*	1	1.2

The most common microbe of CVC infection was *Staphylococcus epidermidis* (35.9%), followed by *Staphylococcus hominis subsp. hominis* (23.1%) and *Staphylococcus aureus* (5.1%). The most common microbe of abdominal-pelvic infection was *Enterococcus faecalis* (24.0%), followed by *Enterobacter cloacae subsp. Cloacae* (16.0%) and *Escherichia coli* (16.0%). The most common microbe of pulmonary infection was *Acinetobacter baumannii* (34.8), followed by *Staphylococcus aureus* (21.7%) and *Pseudomonas aeruginosa* (21.7%) (Table 4).

**Table 4:** The common microbes isolated from the postoperative infection sites of PMP patients

Infection sites	The common microbe 1 (%)	The common microbe 2 (%)	The common microbe 3 (%)
CVC infection	*Staphylococcus epidermidis* (35.9)	*Staphylococcus hominis subsp. Hominis* (23.1)	*Staphylococcus aureus* (5.1)
Abdominal-pelvic infection	*Enterococcus faecalis* (24.0)	*Enterobacter cloacae subsp. Cloacae* (16.0)	*Escherichia coli* (16.0)
Pulmonary infection	*Acinetobacter baumannii* (34.8)	*Staphylococcus aureus* (21.7)	*Pseudomonas aeruginosa* (21.7)
Surgical wound infection	*Staphylococcus epidermidis* (30.0)	*Escherichia coli* (30.0)	*Enterobacter kobei* (10.0)
Urinary system infection	*Escherichia coli* (60.0)	*Candida albicans* (20.0)	*Enterococcus faecalis* (20.0)
Blood culture bacteria/fungi positive (unknown infection site)	*Staphylococcus epidermidis* (40.0)	*Staphylococcus hominis subsp. Hominis* (20.0)	*Klebsiella pneumoniae* (20.0)

*CVC* Central venous catheter

#### The result of antibiotics sensitivity

Among the microbes isolated from the 82 PMP patients with postoperative infections, the most sensitive antibiotics for Gram-positive bacteria were vancomycin (98.4%), linezolid (78.7%) and tigacycline (72.1%). The most sensitive antibiotics for Gram-negative bacteria were levofloxacin (68.5%), amikacin (66.7%) and cefepime (64.8%). The most sensitive antibiotics for fungi were fluconazole (83.3%), voriconazole (83.3%) and flucytosine (50.0%) (Table 5).

**Table 5:** The results of antibiotics sensitivity to microbes isolated from PMP patients with postoperative infection

Types	The results of antibiotics sensitivity test (sensitivity rate)					
Gram-positive bacteria	Vancomycin (98.4%)	Linezolid (78.7%)	Tigecycline (72.1%)	Rifampicin (72.1%)	Quinuptin/Dafoptin (63.9%)	Gentamicin (60.7%)
Staphylococcus epidermidis	Vancomycin (100.0%)	Rifampicin (100.0%)	Linezolid (85.7%)	Tigecycline (85.7%)	Quinuptin/Dafoptin (76.2%)	Gentamicin (71.4%)
Staphylococcus hominis subsp. Hominis	Vancomycin (100.0%)	Rifampicin (100.0%)	Quinuptin/Dafoptin (100.0%)	Gentamicin (80.0%)	Linezolid (70.0%)	Tigecycline (70.0%)
Staphylococcus aureus	Vancomycin (100.0%)	Linezolid (100.0%)	Quinuptin/Dafoptin (90.0%)	Gentamicin (90.0%)	Rifampicin (90.0%)	Sulfamethoxazole (90.0%)
Enterococcus faecalis	Vancomycin (100.0%)	Penicillin G (100.0%)	Ampicillin (100.0%)	Linezolid (77.8%)	Ciprofloxacin (77.8%)	Levofloxacin (77.8%)
Staphylococcus capitis	Vancomycin (100.0%)	Linezolid (100.0%)	Quinuptin/Dafoptin (100.0%)	Rifampicin (100.0%)	Tetracycline (100.0%)	Tigecycline (100.0%)
Gram-negative bacteria	Levofloxacin (68.5%)	Amikacin (66.7%)	Cefepime (64.8%)	Meropenem (63.0%)	Piperacillin (59.3%)	Imipenem (59.3%)
Acinetobacter baumannii	Minocycline (64.3%)	Sulfamethoxazole (50.0%)	Levofloxacin (42.9%)	Meropenem (35.7%)	Gentamicin (35.7%)	Tobramycin (35.7%)
Klebsiella pneumoniae	Amikacin (75.0%)	Imipenem (62.5%)	Meropenem (50.0%)	Aztreonam (50.0%)	Cefepime (50.0%)	Ceftazidime (50.0%)
Escherichia coli	Amikacin (100.0%)	Piperacillin (90.0%)	Imipenem (90.0%)	Ertapenem (90.0%)	Cefepime (80.0%)	Meropenem (80.0%)
Enterobacter cloacae subsp. Cloacae	Amikacin (100.0%)	Ciprofloxacin (100.0%)	Levofloxacin (100.0%)	Cefepime (85.7%)	Gentamicin (85.7%)	Tobramycin (85.7%)
Pseudomonas aeruginosa	Amikacin (100.0%)	Meropenem (100.0%)	Cefepime (100.0%)	Ceftazidime (100.00%)	Ciprofloxacin (100.00%)	Levofloxacin (100.00%)
Fungus	Fluconazole (83.33%)	Voriconazole (83.33%)	Flucytosine (50.00%)	Itraconazole (33.33%)	Amphotericin B (16.67%)	
Candida albicans	Fluconazole (100.00%)	Voriconazole (100.00%)	Flucytosine (33.33%)			

The results of antibiotics sensitivity of common gram-positive bacteria were as follows, *Staphylococcus epidermidis*: vancomycin (100.0%), rifampicin (100.0%), linezolid (85.7%); *Staphylococcus hominis subsp. Hominis*: vancomycin (100.0%), quinuptin/dafoptin (100.0%), gentamicin (80.0%); *Staphylococcus aureus*: vancomycin (100.0%), linezolid (100.0%), quinuptin/dafoptin (90.0%); *Enterococcus faecalis*: vancomycin (100.0%), penicillin G (100.0%), ampicillin (100.0%); *Staphylococcus capitis*: vancomycin (100.0%), linezolid (100.0%), quinuptin/dafoptin (100.0%). The results of antibiotics sensitivity of common gram-negative bacteria were as follows, *Acinetobacter baumannii*: minocycline (64.3%), sulfamethoxazole (50.0%), levofloxacin (42.9%); *Klebsiella pneumoniae:* amikacin (75.0%), imipenem (62.5%), meropenem (50.0%); *Escherichia coli*: amikacin (100.0%), piperacillin (90.0%), imipenem (90.0%); *Enterobacter cloacae subsp. Cloacae*: amikacin (100.0%), ciprofloxacin (100.0%), levofloxacin (100.0%); *Pseudomonas aeruginosa*: amikacin (100.0%), meropenem (100.0%), cefepime (100.0%).

### Specific infectious bacteria

Among the 82 PMP patients with postoperative infection, 6 were infected with multidrug-resistant bacteria (all occurred in pulmonary infection), of which 2 were *Klebsiella pneumoniae* and 4 were *Acinetobacter baumannii*. The antibiotics sensitivity test shows that 2 cases of *Klebsiella pneumoniae* were only sensitive to chloramphenicol and amikacin, respectively. 2 cases of *Acinetobacter baumannii* were all antibiotics-resistant and the other 2 cases were only sensitive to minocycline.

### Correlation and survival analysis of postoperative infection

#### Univariate and multivariate analysis of postoperative infection

Univariate analysis showed that the following factors were associated with postoperative infection: pathological diagnosis (*P* = 0.001), operative duration (*P* = 0.002), number of organs resected (*P* = 0.019), splenectomy (*P* = 0.010), number of stripped peritoneum area (*P* = 0.026), PCI (*P* = 0.004), CC score (*P* = 0.008), intraoperative blood loss (*P* = 0.004), red blood cell transfusion (*P* = 0.002), plasma transfusion (*P* = 0.012), ascites volume (*P* = 0.002) (Table 6).

**Table 6:** The univariate analysis of PMP patients with postoperative infection

variable	Infected (*n* = 82)	Non-infected (*n* = 400)	*P*
Gender, n (%)			0.092
Male	29 (35.4)	182 (45.5)	
Female	53 (64.6)	218 (54.5)	
Age (years), n (%)			0.692
< 60	51 (62.2)	258 (64.5)	
≥ 60	31 (37.8)	142 (35.5)	
Pathological diagnosis, n (%)			**0.001**
Acellular mucus	1 (1.2)	14 (3.5)	
Low grade	60 (73.2)	208 (52.0)	
High grade	20 (24.4)	133 (33.3)	
High grade with signet ring cells	1 (1.2)	45 (11.3)	
Vascular invasion, n (%)			0.103
No	81 (98.8)	374 (93.5)	
Yes	1 (1.2)	26 (6.5)	
Lymphatic metastasis, n (%)			0.094
No	80 (97.6)	370 (92.5)	
Yes	2 (2.4)	30 (7.5)	
Operative duration (min), n (%)			**0.002**
< 680	34 (41.5)	241 (60.3)	
≥ 680	48 (58.5)	159 (39.8)	
Resected organs, n (%)			**0.019**
< 3	23 (28.0)	168 (42.0)	
≥ 3	59 (72.0)	232 (58.0)	
Splenectomy			**0.010**
Yes	40 (48.8)	135 (33.8)	
No	42 (51.2)	265 (66.3)	
Stripped peritoneum area, n (%)			**0.026**
< 6	32 (39.0)	210 (52.5)	
≥ 6	50 (61.0)	190 (47.5)	
Anastomosis, n (%)			0.504
No	20 (24.4)	112 (28.0)	
Yes	62 (75.6)	288 (72.0)	
PCI, n (%)			**0.004**
< 20	13 (15.9)	126 (31.5)	
≥ 20	69 (84.1)	274 (68.5)	
CC, n (%)			**0.008**
0–1	33 (40.2)	225 (56.4)	
2–3	49 (59.8)	174 (43.6)	
Intraoperative blood loss (mL), n (%)			**0.004**
< 350	9 (11.0)	102 (25.5)	
≥ 350	73 (89.0)	298 (74.5)	
RBC transfusion volume (U), n (%)			**0.002**
< 3	28 (34.1)	212 (53.0)	
≥ 3	54 (65.9)	188 (47.0)	
Plasma transfusion volume (mL), n (%)			**0.012**
< 700	25 (30.5)	182 (45.5)	
≥ 700	57 (69.5)	218 (54.5)	
Ascites (mL), n (%)			**0.002**
< 300	19 (23.2)	166 (41.5)	
≥ 300	63 (76.8)	234 (58.5)	

*PCI* Peritoneal cancer index, *CC* Completeness of cytoreduction, *RBC* Red blood cells

The factors above with *P* < 0.05 were incorporated into the binary Logistic regression model, and the results of multivariate analysis showed that intraoperative blood loss ≥ 350 mL (*P* = 0.019) and ascites volume ≥ 300 mL (*P* = 0.008) were independent risk factors for postoperative infection. PMP patients with intraoperative blood loss ≥ 350 mL had a 2.454 times risk of postoperative infection than those with intraoperative blood loss < 350 mL (*P* = 0.019, OR = 2.454, 95%CI: 1.157–5.203); For PMP patients with ascites volume ≥ 300 mL, the risk of postoperative infection was 2.192 times than those with ascites volume < 300 mL (*P* = 0.008, OR = 2.192, 95%CI: 1.233–3.897) (Table 7).

**Table 7:** The multivariate analysis of PMP patients with postoperative infection

Variable	Wald	OR	95%CI	*P*
Intraoperative blood loss (mL)				
≥ 350 *vs*. < 350	5.482	2.454	1.157–5.203	0.019
Ascites volume (mL)				
≥ 300 *vs*. < 300	7.148	2.192	1.233–3.897	0.008

*OR* Odds ratio; *CI* Confidence interval

#### Survival analysis

**Fig. 1:**
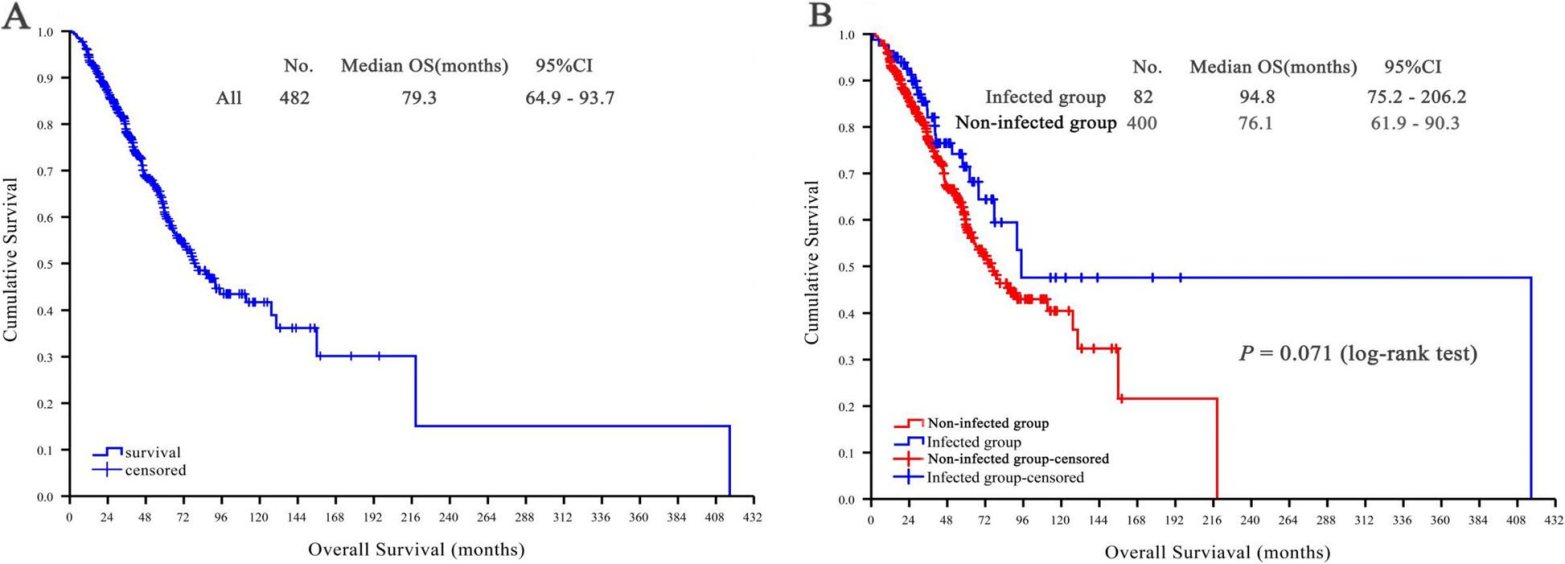
Survival analysis. **A** Overall survival analysis of all PMP patients; (**B**) Survival curve analysis of infected group and non-infected group

Among 482 PMP patients, the median follow-up was 56.1 (95%CI: 51.6–60.6) months. There were 173 (35.9%) patients died, and 309 (64.1%) survived, with median OS of 79.3 (95%CI: 64.9–93.7) months (Fig. 1A). There was no significant difference in median OS between the infected and non-infected groups (76.1 vs.94.8 months, *P* = 0.071) (Fig. 1B).

## Discussion

In this study, the infection rate of PMP patients after CRS + HIPEC was about 17.0%. The most common infection was CVC infection (8.1%), followed by abdominal-pelvic infection (5.2%) and pulmonary infection (4.8%). Antibiotics sensitivity test revealed vancomycin as the most sensitive antibiotic for Gram-positive bacteria (98.4%), levofloxacin as most sensitive antibiotic for Gram-negative bacteria (68.5%), and fluconazole as the most sensitive antibiotic for fungi (83.3%). Univariate and multivariate analysis revealed that ascites volume ≥ 300 mL and intraoperative blood loss volume ≥ 350 mL were independent risk factors for postoperative infection.

CRS + HIPEC is the standard treatment for PMP, which can significantly improve the survival of patients with acceptable safety [11, 12]. However, PMP patients treated with CRS + HIPEC usually had received several operations and multicycle chemotherapy. Most of these patients have poor physical condition and are at high risk of adverse events after invasive multi-organ resection such as CRS [22]. In addition, HIPEC drugs can not only kill residual tumor cells in patients' abdominal cavity, but also have drug toxicity and immunosuppressive effects, making PMP patients potentially high-risk for postoperative infection [23, 24].

Previous studies have shown that the infection rate of PM patients after CRS + HIPEC was about 21.0% ~ 43.0% [15–19, 25]. The study of Arslan et al. [22] on 169 PM patients showed that the postoperative infection rate of CRS + HIPEC was 27.8%, and the most common was surgical site infection (21.3%). Smibert et al. [19] analyzed 100 patients treated with CRS + HIPEC, and the results showed that the postoperative infection rate was 43.0%, with surgical site infection being the most common (27.0%). At our center, the postoperative infection was most frequently observed in the colorectal cancer peritoneal metastases (24.3%), and least common in PMP (17.0%) (Table 8). The overall postoperative infection rate among PM patients was 20.1% which was lower than the postoperative infection rate reported in previous studies (Table 9). This could be attributed to the mature CRS + HIPEC treatment system of our center (the center has successfully completed more than 2000 CRS + HIPEC operations so far). Each PM patient will receive adequate preoperative preparation, lung function exercise, and enteral or intra intestinal nutrition support according to the nutritional status of the patient.

**Table 8:** The comparison of postoperative infection rate after CRS + HIPEC for different PM patients at our center

PM	Patients number	Number of infected patients	Postoperative infection rate (%)
Gastric cancer PM	114	21	18.4
Colorectal cancer PM	301	72	24.3
Pseudomyxoma peritonei	482	82	17.0
Malignant peritoneal mesothelioma	177	32	18.1
Ovarian cancer PM	279	65	18.3
Total	1353	272	20.1

*PM* Peritoneal metastases

**Table 9:** The literatures of infection after CRS + HIPEC in PM Patients

Authors	Year	No	Postoperative infection rate (%)	The most common infection site (%)	The most common microbe
Capone, et al. [26]	2007	30	36.7	Surgical site	*Candida albicans*
Mizumoto, et al. [16]	2012	250	23.6	Surgical site	NA
Haslinger, et al. [15]	2013	112	34.0	Surgical site	NA
Valle M, et al. [18]	2014	111	35.8	Surgical site	*Staphylococcus epidermidis*
Arslan, et al. [22]	2017	169	27.8	Surgical site	*Escherichia coli*
Smibert, et al. [19]	2019	100	43.0	Surgical site	*Escherichia coli*
Cardi, et al. [25]	2019	200	21.0	Surgical site	*Candida albicans*
Viyuela García, et al. [17]	2020	112	25.4	Surgical site	NA
This study	2023	482	17.0	CVC	*Staphylococcus epidermidis*

*CVC* Central venous catheter. Surgical site: including abdominal-pelvic infection and surgical wound infection

Previous studies also identified major microbial pathogens for postoperative infection. Arslan et al. [22] analyzed 47 infected patients after CRS + HIPEC, and the microbe culture results showed that *Escherichia coli* (47.1%) was the most common bacteria. Valle et al. [18] studied 78 patients infected after CRS + HIPEC and found that the most common bacteria infected was *Staphylococcus epidermidis* (16.7%). In this study, the most common postoperative infection of PMP patients was *Staphylococcus epidermidis* (25.6%). Meanwhile, according to the antibiotic sensitivity test, the most common microbe infection of CVC was *Staphylococcus epidermidis* (16.7%), which was highly sensitive to vancomycin, linezolid, tigecycline and so on. *Enterococcus faecalis* (9.8%) was the most common microbe isolated from abdominal-pelvic infection, with high sensitivity to vancomycin, penicillin G, ampicillin. The most common microbe isolated from pulmonary infection was *Acinetobacter baumannii* (15.9%), while minocycline and sulfamethoxazole were the only relatively sensitive antibiotics. In this study, 6 cases of PMP patients infected with multi-drug resistant bacteria, all of which were pulmonary infections, suggesting that after CRS + HIPEC, it is especially necessary to pay much attention to the pulmonary function of patients, promote sputum discharge and reduce the risk of pulmonary infection. Some studies had shown that [22, 26], the main cause of death due to infection in patients after CRS + HIPEC is *Candida albicans* infection. In this study, none of PMP patients died or became critically ill due to *Candida albicans* infection, and antibiotics tests showed that *Candida albicans* was very sensitive to fluconazole and voriconazole.

There were several studies about postoperative infection of CRS + HIPEC revealed the following 5 major risk factors, including colorectal resection, small intestine resection, intraoperative blood loss, operation duration > 10 h, and preoperative nutritional status [18, 19, 25]. In comparison, our study only found 2 independent risk factors for postoperative infection, intraoperative blood loss ≥ 350 mL and ascites volume ≥ 300 mL. Patients with ascites tended to have abdominal distension, poor appetite and other gastrointestinal symptoms, and the nutritional status of such patients were usually poor. However, nutritional status had been shown to have a significant impact on the immune system, and patients with impaired immune response were more likely to develop postoperative complications after gastrointestinal surgery [20, 27]. There was a bacteria hypothesis in the mucin formation and tumor progression in PMP, Semino-Mora et al. [28]. found that the overall bacterial density of appendixes in PMP patients was much higher than in healthy people. That is probably an explanation to why ascites is associated with the infection risk, because ascites could be produced partially by bacteria. CRS + HIPEC often involved partial resection of the invaded gastrointestinal tract, for which ERAS guidelines recommend preoperative mechanical gastrointestinal preparation with or without oral antibiotics to reduce postoperative infection rates [29].

Postoperative infection was the main cause of increased length of stay and perioperative mortality in patients treated with CRS + HIPEC, as well as increased treatment costs for patients [8, 30]. However, no studies have shown that postoperative infection of CRS + HIPEC was associated with long-term outcome of patients. The results of survival analysis in this study also showed that there had no difference on median overall survival between PMP patients with or without postoperative infection.

This study has the following limitations: First, the types and characteristics of microbes isolated from the infected patients may be different in different CRS + HIPEC treatment centers, so it is necessary to combine infection-related data from multiple centers in the future to make a summary. Second, this was a single-center retrospective case–control study with a moderate sample size, and higher-level studies must verify the conclusions.

In conclusion, PMP patients may have increased infection risk after CRS + HIPEC, especially CVC, abdominal-pelvic and pulmonary infections. This study analyzed the common infection sites and microbes in PMP patients after CRS + HIPEC, as well as the corresponding antibiotics sensitivity test results, which may provide reference for the early clinical empirical antibiotics use in patients with such CRS + HIPEC postoperative infection.

## Data Availability

The datasets used and/or analyzed during the current study are available from the corresponding author on reasonable request.

## References

[1] Carr NJ, Cecil TD, Mohamed F (2016). A consensus for classification and pathologic reporting of pseudomyxoma peritonei and associated appendiceal neoplasia: the results of the Peritoneal Surface Oncology Group International (PSOGI) modified Delphi process. Am J Surg Pathol.

[2] Smeenk RM, Bruin SC, van Velthuysen ML (2008). Pseudomyxoma peritonei. Curr Probl Surg.

[3] Smeenk RM, van Velthuysen ML, Verwaal VJ (2008). Appendiceal neoplasms and pseudomyxoma peritonei: a population based study. Eur J Surg Oncol.

[4] Mittal R, Chandramohan A, Moran B (2017). Pseudomyxoma peritonei: natural history and treatment. Int J Hyperthermia.

[5] Yang R, Su YD, Ma R (2023). Clinical epidemiology of peritoneal metastases in China: The construction of professional peritoneal metastases treatment centers based on the prevalence rate. Eur J Surg Oncol.

[6] Hinson F, Ambrose N (1998). Pseudomyxoma peritonei. Br J Surg.

[7] Wang H, Wang X, Ju Y (2014). Clinicopathological features and prognosis of pseudomyxoma peritonei. Exp Ther Med.

[8] Elias D, Gilly F, Quenet F (2010). Pseudomyxoma peritonei: a French multicentric study of 301 patients treated with cytoreductive surgery and intraperitoneal chemotherapy. Eur J Surg Oncol.

[9] Guaglio M, Sinukumar S, Kusamura S (2018). Clinical surveillance after macroscopically complete surgery for low-grade appendiceal mucinous neoplasms (LAMN) with or without limited peritoneal spread: long-term results in a prospective Series. Ann Surg Oncol.

[10] Ansari N, Chandrakumaran K, Dayal S (2016). Cytoreductive surgery and hyperthermic intraperitoneal chemotherapy in 1000 patients with perforated appendiceal epithelial tumours. Eur J Surg Oncol.

[11] Li Y, Xu HB, Peng Z (2019). Expert consensus on cytoreductive surgery plus hyperthermic intraperitoneal chemotherapy in the treatment of pseudomyxoma peritonei. Natl Med J Chin.

[12] Govaerts K, Lurvink RJ, De Hingh IHJT (2021). Appendiceal tumours and pseudomyxoma peritonei: literature review with PSOGI/EURACAN clinical practice guidelines for diagnosis and treatment. Eur J Surg Oncol.

[13] Yang R, Su YD, Liu G (2023). Effect of standardized fluid management on cardiac function after CRS + HIPEC in patients with PMP: a single-center case-control study. Int J Hyperthermia.

[14] Deslauriers N, Olney H, Younan R (2011). Splenectomy revisited in 2011: Impact on hematologic toxicities while performing cytoreductive surgery and hyperthermic intraperitoneal chemotherapy. J Gastrointest Oncol.

[15] Haslinger M, Francescutti V, Attwood K (2013). A contemporary analysis of morbidity and outcomes in cytoreduction/hyperthermic intraperitoneal chemoperfusion. Cancer Med.

[16] Mizumoto A, Canbay E, Hirano M (2012). Morbidity and mortality outcomes of cytoreductive surgery and hyperthermic intraperitoneal chemotherapy at a single institution in Japan. Gastroenterol Res Pract.

[17] Viyuela García C, Medina Fernández FJ, Arjona-Sánchez Á (2020). Systemic inflammatory markers for the detection of infectious complications and safe discharge after cytoreductive surgery and HIPEC. Surg Oncol.

[18] Valle M, Federici O, Carboni F (2014). Postoperative infections after cytoreductive surgery and HIPEC for peritoneal carcinomatosis: proposal and results from a prospective protocol study of prevention, surveillance and treatment. Eur J Surg Oncol.

[19] Smibert OC, Slavin MA, Teh B (2020). Epidemiology and risks for infection following cytoreductive surgery and hyperthermic intra-peritoneal chemotherapy. Support Care Cancer.

[20] Skeie E, Koch AM, Harthug S (2018). A positive association between nutritional risk and the incidence of surgical site infections: a hospital-based register study. PLoS One.

[21] Yang XJ, Huang CQ, Suo T (2011). Cytoreductive surgery and hyperthermic intraperitoneal chemotherapy improves survival of patients with peritoneal carcinomatosis from gastric cancer: final results of a phase III randomized clinical trial. Ann Surg Oncol.

[22] Arslan NC, Sokmen S, Avkan-Oguz V (2017). Infectious complications after cytoreductive surgery and hyperthermic intra-peritoneal chemotherapy. Surg Infect (Larchmt).

[23] Canda AE, Sokmen S, Terzi C (2013). Complications and toxicities after cytoreductive surgery and hyperthermic intraperitoneal chemotherapy. Ann Surg Oncol.

[24] Glehen O, Osinsky D, Cotte E (2003). Intraperitoneal chemohyperthermia using a closed abdominal procedure and cytoreductive surgery for the treatment of peritoneal carcinomatosis: Morbidity and mortality analysis of 216 consecutive procedures. Ann Surg Oncol.

[25] Cardi M, Sibio S, Di Marzo F (2019). Prognostic factors influencing infectious complications after cytoreductive surgery and hipec: Results from a tertiary referral center. Gastroenterol Res Pract.

[26] Capone A, Valle M, Proietti F (2007). Postoperative infections in cytoreductive surgery with hyperthermic intraperitoneal intraoperative chemotherapy for peritoneal carcinomatosis. J Surg Oncol.

[27] Hennessey DB, Burke JP, Ni-Dhonochu T (2010). Preoperative hypoalbuminemia is an independent risk factor for the development of surgical site infection following gastrointestinal surgery: a multi-institutional study. Ann Surg.

[28] Semino-Mora C, Liu H, McAvoy T (2008). Pseudomyxoma peritonei: is disease progression related to microbial agents? A study of bacteria, MUC2 AND MUC5AC expression in disseminated peritoneal adenomucinosis and peritoneal mucinous carcinomatosis. Ann Surg Oncol.

[29] Hübner M, Kusamura S, Villeneuve L (2020). Guidelines for Perioperative Care in Cytoreductive Surgery (CRS) with or without hyperthermic IntraPEritoneal chemotherapy (HIPEC): Enhanced recovery after surgery (ERAS®) Society Recommendations - Part I: Preoperative and intraoperative management. Eur J Surg Oncol.

[30] Chua TC, Saxena A, Schellekens JF (2010). Morbidity and mortality of cytoreductive surgery and perioperative intraperitoneal chemotherapy at a single tertiary institution. Ann Surg.

